# Improving the Integrated Fabrication of Insulation Systems in Electric Drives by Injection Molding of Thermosets Due to Processing Conditions and Slot Design

**DOI:** 10.3390/polym15051165

**Published:** 2023-02-25

**Authors:** Uta Rösel, Maximilian Kneidl, Jörg Franke, Dietmar Drummer

**Affiliations:** 1 Institute of Polymer Technology, Friedrich-Alexander-Universität Erlangen-Nürnberg, 91058 Erlangen, Germany; 2 Institute for factory Automation and Production Systems, Friedrich-Alexander-Universität Erlangen-Nürnberg, 91058 Erlangen, Germany

**Keywords:** thermoset, injection molding, insulation system

## Abstract

The expanding demand for electro mobility in general and specifically for electrified vehicles requires the expansion of electro mobility technology with respect to variations in the requirements of the process and the application. Within the stator, the electrical insulation system has a high impact on the application properties. So far, limitations, such as the identification of suitable materials for the stator insulation or high costs in the processes, have hindered the implementation of new applications. Therefore, a new technology that allows integrated fabrication via the injection molding of thermosets is founded in order to expand the applications of stators. The possibility of the integrated fabrication of insulation systems to meet the demands of the application can be improved by the processing conditions and the slot design. Within this paper, two epoxy (EP) types with different fillers are investigated to show the impact of the fabrication process in terms of different parameters; these include the holding pressure or the temperature setup, as well as the slot design and with that the flow conditions. To evaluate the improvement in the insulation system of electric drives, a single slot sample, consisting of two parallel copper wires, was used. Then, the two parameters of the average partial discharge (PD) and the partial discharge extinction voltage (PDEV), as well as the full encapsulation detected by microscopy images, were analyzed. It was shown that both characteristics (electric properties—PD and PDEV; full encapsulation) could be improved in terms of an increase in the holding pressure (up to 600 bar) or a reduction in the heating time (around 40 s), as well as the injection speed (down to 15 mm/s). Further, an improvement in the properties can be reached by increasing the space between the wires, as well as the wire and the stack, due to a higher slot depth or by implementing flow-improving grooves that have a positive effect on the flow conditions. With that, the optimization of the integrated fabrication of insulation systems in electric drives via the injection molding of thermosets was enabled with respect to the process conditions and the slot design.

## 1. Introduction

The development of electric drive technology has significantly expanded due to its rising demand within the electro mobility sector and, more specifically, in terms of electrified vehicles or powertrains. This goes along with a changing requirement not only in terms of the product, but also in terms of the production process. For example, a high level of automation in the production process, alongside low rejection rates, is needed to meet the increasing demand for reduced manufacturing costs and increase the economic efficiency. With respect to the properties of the product, a high-power density and an optimum efficiency should be realized in the electric drive unit, along with low weight and little installation space [1]. The stator insulation system determines the power output of the motor, where thermal and electrical limitations have to be considered during the whole life cycle [2]. Therefore, the electrical insulation system occupies a special role within the development of electromobility applications.

The classic stator insulation system can be divided into two groups of insulation: primary insulation, covering the conductors, and secondary insulation, found in the slot and in the full stator via impregnation. The basic form of electrical insulation is realized by the insulation of the conductors and avoids electrical flashovers between wires; this causes a reduction in the performance. Commonly, the insulation of the conductors is covered with Polyamide-imide (PAI) varnishes or Polyimide (PI) foils. To ensure sufficient insulation, the conductor is swathed many times with the foils. Further, high temperature polymers, such as Polyether ether ketone (PEEK) or Polyphenylene sulfide (PPS), are used to fulfill the increasing demand for applications within the automotive or aerospace industries [3]. The slot ground insulation enlarges the size of the conductor lagging against the laminated core of the stator further and ensures the protection of the primary insulation from damage due to the insertion of the coils. Therefore, flat insulating materials, such as foils made out of Polyester imides or aramides, as well as inorganic materials such as mica, are used within a multi-layer structure [2]. The final insulation step provides the fixture of the windings in their position by impregnation using an epoxy (EP) or unsaturated polyester resin, for example. This final step occupies a huge proportion of the total time taken to produce the stator. It ensures, however, the protection of the windings in a mechanical and chemical way and allows the dissipation of heat. This heat is generated in the conductor due to losses during operation, which means that it is important to find a way to transfer this stored heat [4]. The methods of the final insulation step can be categorized into impregnation and casting, which uses processing under a vacuum, under an atmosphere or overpressure, as well as a combination of them. The second group of casting methods sees the full encapsulation of the stator, as the windings and the winding heads are completely enclosed with resin. The method by casting leads to a higher quality insulation system compared to impregnation, due to the fewer defects. However, the casting method also has disadvantages in terms of its processing time and its material consumption.

The impregnation of the stator windings can be further divided into the step of insulation by liquid resin and the thermal curing process. The first step is subdivided into dip, roller and trickle impregnation. Within the dip and roller impregnation process, the resin infiltrates the winding within the preheated stator, after the stator is placed into a soaking tank. The high temperature of the preheated stator ensures that the resin slowly reaches the gelation point and cures. After the curing, the excess material has to be removed. With respect to the resin and the mechanism of the curing, the process can be accelerated by applying an additional current to the windings or by using UV lamps [5]. In the trickle impregnation process, the resin is poured onto the winding heads via an individual nozzle and by using the capillary effect, which forces the resin into the bottom of the slots in order to impregnate the entire stator. This significantly reduces the material impact compared to the dip or roller-based processes. However, the methods of soaking reveal a higher possibility of deficiency due to trapped air, and with that, a reduced insulation strength in the system [5]. The different methods of impregnation reveal several challenges in terms of ensuring that there is efficient technology with respect to large-scale production. At the moment, the main limitations of the process are the long cycle times of the semi-finished products and the curing of the resin, as well as a lack of modification to the material behavior in terms of the thermal conductivity and mechanical of the application.

The production of the stator is divided into the following steps: the production of the laminated core, the slot base insulation, the winding of the coils, the impregnation of the windings and the contacting of the conductors [6]. The impregnation of the stator is normally carried out using trickle impregnation via applications in the automotive industry. This entails long process times because of the curing, the low reproducibility of the samples because of trapped air, which is likely to occur, and the low thermal conductivity of the resins, which is needed to reduce the viscosity of the processing method [1]. These limitations of the impregnation process can be expanded using a different fabrication method: thermoset injection molding. Within this production step, the thermoset resin is implemented into a low-temperature screw unit where the plastification and the homogenization takes place. The material is injected into the cavity, which reveals a high temperature. Due to the increase in the temperature, the viscosity of the thermoset drops abruptly. This reply is overlapped by the irreversible crosslinking reaction, which causes the route of the viscosity to increase again [7]. This process allows the application of pressure during injection and curing as the mold itself is closed. The applied holding pressure significantly reduces defects such as trapped air and allows the attainment of a repeatable component weight [8]. Further, the process of injection molding itself allows the process to reach minor cycle times, improved conditions within the handling of the material and material modification that is relative to the demand of the application [7]. A granulated thermoset is used within the injection molding process; this is based on a premixed resin hardener system with up to 85 mass-% of fillers. Because of the application of these fillers, a wide range of modifications to the material behavior is possible. For example, the thermal conductivity of thermosets can be increased from 0.2 $\frac{W}{m\cdot K}$ to over 2.5 $\frac{W}{m\cdot K}$ [9].

The complete impregnation of the electrical conductors by the thermoset has to be ensured in order to use the process of thermoset injection molding and realize the insulation of stators. Impregnation is defined as the flow of a liquid through a porous medium [10]. In the case of the stator, the porous medium is represented by the windings and the slots between the conductors. With respect to [11], the flow path of the liquid, or more precisely, its impregnability in terms of a two-dimensional perspective, is influenced by the viscosity of the liquid, the permeability of the porous medium, the impregnation time and the processing pressure. These impact factors can be adapted to the injection molding process, with the exception of the viscosity of the liquid. This exception is based on the fact that a thermoset molding compound is not a Newtonian fluid, as assumed by [11]. Ref. [12] proved the complete impregnation of conductor-like structures based on carbon rovings due to the injection molding process with highly filled thermosets. It was demonstrated that several process parameters directly influence the impregnation quality. Temperature, for example, is a major factor that reduces the viscosity; this accompanies an improvement in the impregnation and the realization of a complete insulation, analogous to [11]. The general usage of the injection molding process in order to fabricate a stator is already realized to some extent with respect to [13,14,15]. However, only stators with small dimensions and short flow paths or stator segments have been implemented so far.

The aim of this paper is to show the different options that might improve the insulation systems of electric drives by injection molding, in order to enable the application of insulation systems in drive technologies that are fabricated by the injection molding process to a broader extend. Two main routes of enhancement were analyzed, one regarding the process conditions and one with respect to the geometry of the slot. With respect to the process parameters, the mold temperature, the holding pressure, the heat time and the injection velocity were changed to mainly influence the viscosity of the resin in the cavity with respect to the time and temperature-dependent rheology behavior. The concept aimed to reach a low viscosity within the cavity to ensure full insulation between the wires and the metal stack, as well as between the two wires in order to improve the properties with respect to its application in electric drives. Further, the insulation should have been improved by increasing the space between the wires within the metal stack in order to increase the possibility of the resin flowing between the wires. Therefore, the depth of the wire slot was changed. In addition, a more complex slot structure was investigated, in which a groove was implemented; this was meant to force the material to flow primarily along this path to fill the provided pockets and thus implement resin between the wire and the metal stack. With respect to the application, the average partial discharge should have reached a low and the partial discharge extinction voltage should have reached a high value. Thus, the insulation between the wires, as well as between the wire and the metal stack, was analyzed by microscopy images along the flow path, in order to investigate the possible flow length, at which point the insulation can be reached.

## 2. Materials and Methods

### 2.1. Material

The experiments were based on two commercial types of epoxy resin (EP): the type EP 3162 EMG (Raschig GmbH, Ludwigshafen, Germany) and the type XW 6640-1 (Duresco GmbH, Witterswil, Swizerland). The two resins differ in terms of their anorganic filler material. Both materials are a premixed grey-black granulate; this included a resin, hardener, catalyst and some carbon black pigments. The characterization of the pure filler systems is unfortunately not possible due to the business secret, which accompanies the fact that the exact composition of the mixture, as well as the filler type and grade, is confidential. To understand the influence of the fillers within the material, the basic characterization was compared to a pure epoxy resin without fillers of the type EP 3681 E (Raschig GmbH, Ludwigshafen, Germany). This type relies on the same resin, hardener and catalyst as the other two types. An extensive characterization of the material—additionally in comparison to the unfilled epoxy resin EP 3681 E—can be found in [16], where the impact of the different filler systems is discussed in detail.

EP 3162 EMG has a density of 1.77 $\frac{g}{{\mathrm{cm}}^{3}}$ and XW 6640-1 has a density of 2.41 $\frac{g}{{\mathrm{cm}}^{3}}$. The basic epoxy type EP 3681 E has a density of 1.225 $\frac{g}{{\mathrm{cm}}^{3}}$. The specific heat capacity c is reduced in terms of the fillers, as the basic epoxy type EP 3681 E reaches 1.616 $\frac{J}{g\cdot K}$; meanwhile, EP 3162 EMG reaches 0.997 $\frac{J}{g\cdot K}$ and XW reaches 6640-1 0.899 $\frac{J}{g\cdot K}$. The further characterization of the material is revealed in the datasheets of the materials [17,18].

### 2.2. Fabrication of the Test Specimens

**Figure 1:**
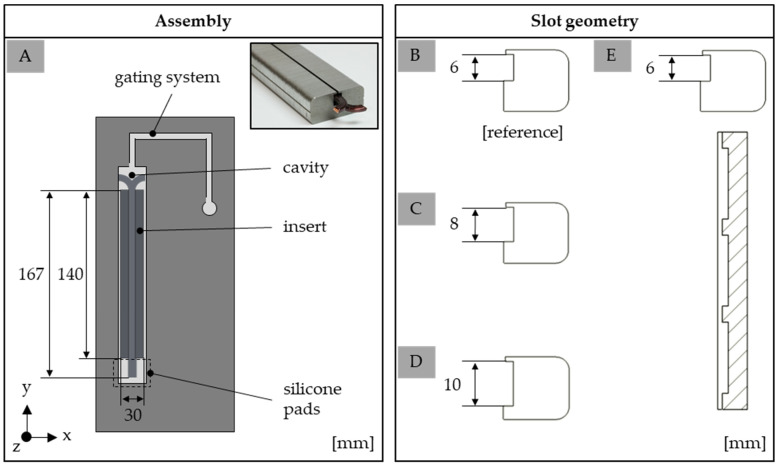
Schematic assembly of the tool to fabricate the single slot sample (**A**) and detailed dimension of the different slot geometries (**B**–**E**).

The test samples were produced using a Krauss Maffei KM 80-380 CX DUR/03 injection molding machine (KraussMaffei Group, Munich, Germany) with a screw diameter of 30 mm. The process was pressure controlled. The test samples—so-called single slot samples—were an assembly, as shown in Figure 1A, where one stack of metal sheets together with two wires are inserted into the tool before the injection molding process starts. To ensure accessible terminals, the cavity is sealed on the opposite side of the gate by using silicone pads. Figure 1 further shows the dimension of the single slot sample in terms of the reference sample. As the design of the slot was one of the factors that was changed to improve the insulation system, Figure 1 shows the three different slot geometries with different slot depths (B–D) and an integrated flow-improving groove (E). The change in the slot depth refers to an increase in the cross section of 30 and 60% compared to the reference.

The processing parameters were set as shown in Table 1 [column 0] in terms of the reference sample. The processing parameters were the second factor, which was analyzed in terms of its influence on the insulation system. Table 1 further portrays the different parameters of the processing conditions, where the mold temperature, the holding pressing and heating time, as well as the injection speed, were considered in terms of an improvement in the insulation system. Each parameter was changed separately, so that only one parameter was different compared to the reference sample.

### 2.3. Characterization

To assess the behavior of the material within the insulation system, the material itself was characterized in terms of the process-relevant properties. Further, the behavior of the single slot samples was analyzed in terms of the insulation properties by evaluating the partial discharge (PD) and the partial discharge extinction voltage (PDEV); it was also evaluated in terms of the visual detection of the insulation between the wires, and the wire and the metal stack, by microscopy images.

#### 2.3.1. Differential Scanning Calorimetry (DSC) According to ISO 11357

To investigate the temperature-dependent reaction kinetics in terms of the process conditions of the material, a differential scanning calorimetry instrument (DSC Q100; TA Instruments, New Castle, DE, USA) was used. Samples of about 5 mg were placed in DSC aluminum pans and heated with a constant rate of 10 K per minute from 0 °C to 240 °C. The experiments were conducted in a nitrogen atmosphere with a flow rate of 50 mL per minute. In addition to the specific enthalpy ΔH_ges;1_ and the peak temperature T_peak_, the reaction turnover α was determined with respect to Equation (1), where ΔH_j_ is the specific enthalpy at the temperature T_j_ and ΔH_ges;1_ is the total specific enthalpy in the first heating cycle [7].

(1) $$\alpha =\left(\frac{{\Delta H}_{j}}{{\Delta H}_{\mathrm{ges};1}}\right)*100\;[\%]$$

#### 2.3.2. Determination of the Viscosity Using a Rotational Viscometer According to DIN EN 6043

In addition to the reaction kinetics, the temperature and time-dependent viscosity defines the material behavior within the processing. A rotational viscometer (Discovery Hybrid Rheometer 2; TA Instruments, New Castle, DE, USA) was used along DIN EN 6043, where the viscosity was determined with respect to an increasing temperature (dynamic behavior) and to the time dependence (isothermal). The assembly was based on two plates with a rotating shearing load and a constant frequency of 1 Hz. In case of dynamic measurements, the temperature range was set between 90 °C and 200 °C, with a constant heat rate of 5 °C per minute. The minimum of the viscosity η_min_ and the corresponding temperature Tη_min_ were defined.

The isothermal plateau for the time-dependent behavior was set at the temperature of η_min_, which was 120 °C in case of EP 3162 EMG and 110 °C for XW 6640-1; this was further increased in steps of 20 °C up to 180 °C, ongoing from 120 °C. The time t_pot_ between the beginning of the calculation and the turning point—the so-called pot life—was analyzed.

#### 2.3.3. Average of Partial Discharge (PD) and Partial Discharge Extinction Voltage (PDEV)

**Figure 2:**
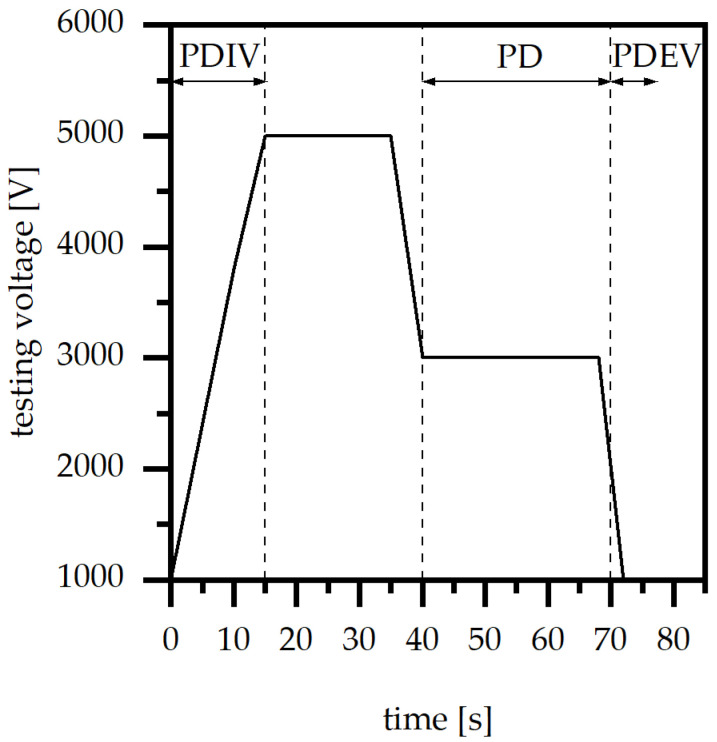
Testing voltage for the measurement of the average of partial discharge (PD), the partial discharge inception (PDIV) and the extinction voltage (PDEV).

The evaluation of the mean partial discharge level (PD) and the partial discharge extinction voltage (PDEV) was realized using test equipment with two parallel electrodes, which is defined by IEC 60243-1, and a measuring system within a Faraday cage according to IEC 60270 (type: Omicron MPD 600; Omicron electronics GmbH, Klaus, Austria). The test samples were produced according to IEC 60034-18-42. The clamps were placed at the end of the two wires, in order to characterize the electric properties of the wire-to-wire insulation system, and also on the batch of the two wire ends and on the stack of metal sheets, in order to analyze the electric properties the wire-to-stack insulation system. After clamping the test samples within the test setting, the values of the partial discharge level (PD), the partial discharge inception (PDIV) and the extinction voltage (PDEV) were defined using the testing profile, as shown in Figure 2. Within this paper, only PD and PDEV are analyzed. The PD threshold for computing the partial discharge inception and extinction voltage was set to 150 pC. The testing time for the partial discharge level (PD) was set to 30 s. Further, the voltage incline was 240 V per second. The experiments were held at a room temperature of 20 °C with a humidity of 50%.

#### 2.3.4. Microscopy

In order to analyze the position of the wire within the stack and the insulation of the wire due to the injection molding process, small strips of the single slot sample were removed using a water-cooled saw with minimal temperature input. The strips were taken from a position near and far away from the gate, as well as from the middle of the flow path, in order to evaluate the change in the position of the wires and the polymer along the flow path. The strip samples were embedded in cold-curing epoxy resin (type: Epofix; Struers GmbH, Ottensoos, Germany) and polished.

Afterwards, the samples were characterized by a reflected light microscope (type: Axio Imager.M2; Carl Zeiss AG, Oberkochen, Germany) and the area of air between the wires was defined based on the images taken.

## 3. Results

### 3.1. Differential Scanning Calorimetry (DSC) According to ISO 11357

**Figure 3:**
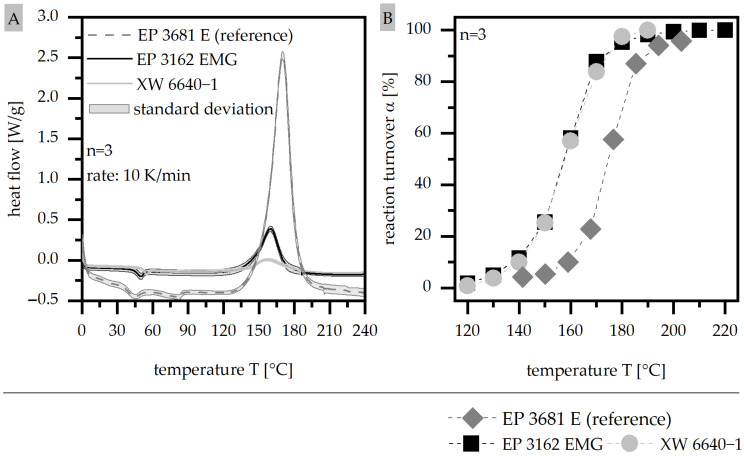
Route of DSC measurements (**A**) and reaction turnover α (**B**) compared for two EP types and the reference material.

The route of the DSC measurement (A) and the reaction turnover α (B) is shown in Figure 3 for the two EP types (EP 3162 E and XW 6640-1) in comparison with the reference material of the pure epoxy resin EP 3681 E. The specific enthalpy ΔH_ges;1_ is significantly reduced in terms of the filled epoxy types compared to the reference, as EP 3162 E needs only 42% and XW 6640-1 needs only 16% of the ΔH_ges;1_ of the reference. This reduction accompanies the implementation of the filler, as they do not participate in the curing process. Further, there is a slight shift in the peak temperature T_peak_, from 10 °C to lower temperatures for the filled systems. The reaction turnover α is similar in the two filled systems but is shifted to lower temperatures compared to the reference. With that, a higher degree of curing takes place in the filled system at a certain temperature, which shifts the curing process to lower temperatures. With respect to the mold temperatures of 170 °C and 190 °C, the reaction kinetics are slightly above T_peak_; α is greater than 80% in the case of 170 °C, and at the offset temperature of the DSC measurement, α is around 100% in the case of 190 °C. With that, the two mold temperatures depict the middle and the end section of the curing process with respect to the reaction kinetics.

### 3.2. Determination of the Viscosity Using a Rotational Viscometer According to DIN EN 6043

**Figure 4:**
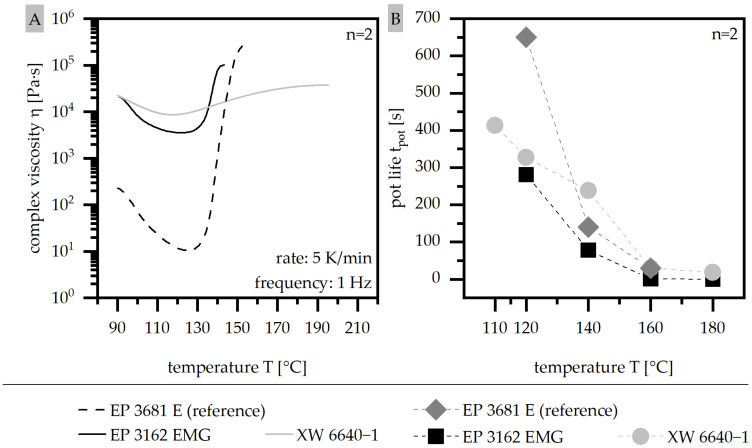
Route of complex viscosity η (dynamic measurement) (**A**) and pot life t_pot_ (isothermal measurement) (**B**) compared for two EP types and the reference material.

The route of the complex viscosity η out of the dynamic measurement (A) and the pot life t_pot_ from the isothermal measurement (B) are shown in Figure 4 for the two filled material systems, together with the unfilled reference material. Due to the filling of the material, η increases and reaches approximately two decades higher values in terms of η_min_ for EP 3162 E and XW 6640-1, compared to EP 3681 E. Further, there is a slight temperature shift of about 5 °C for the filled systems towards the lower temperatures at which η_min_ is reached. Within the filled systems, XW 6640-1 reaches a slightly higher η_min_, which indicates a poorer flowability. However, the level of η_min_ and the temperature at which η increases significantly is shifted to higher temperatures compared to EP 3162 E. For EP 3162 E, the massive increase in η takes place within the temperature range of 130 °C and 140 °C, whereas for XW 6640-1, this increase extends between 130 °C and 180 °C. This allows XW 6640-1 to keep a quite low viscosity over a long period of time.

The pot life t_pot_ reveals a similar behavior in the materials, with a slight reduction in time for the filled system compared to the reference material for temperatures above 140 °C. Under 140 °C, the reference material reaches significantly higher values for t_pot_.

### 3.3. Average of Partial Discharge (PD) and Partial Discharge Extinction Voltage (PDEV)

To assess the behavior of the single slot sample in terms of the insulation system, the averages of the partial discharge (PD) and the partial discharge extinction voltage (PDEV) were analyzed, with respect to the influence of the different process parameters and the variation in the slot geometry. The influence of each parameter onto PD and PDEV is discussed in detail in the following section and summarized afterwards. Further, the improvement in the insulation system between the two wires and between the wire and the metal stack is compared.

#### 3.3.1. Mold Temperature

**Figure 5:**
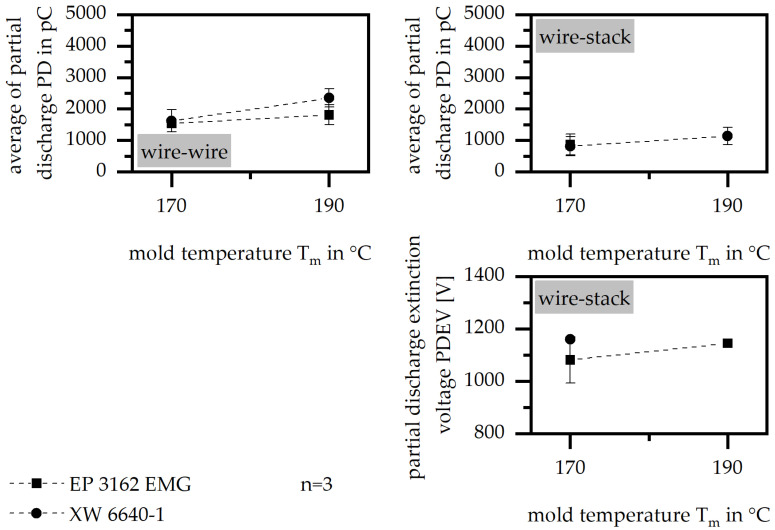
Average of partial discharge (PD) and partial discharge extinction voltage (PDEV) relative to the mold temperature in the insulation system of wire to wire and wire to stack.

The influence of the mold temperature onto the insulation system, as shown in Figure 5, is similar in the wire–wire and the wire–stack system. PD is reduced with a lower mold temperature, which suggests that there is a better separation between the stack and the wire respective of the two wires. Further, the lower mold temperature shifts the curing to a later stage. PDEV is hardly affected, so the mold temperature should be chosen with a low value in order to give the material as much time as possible to insulate within the single slot sample and to improve the flowability via a low viscosity. The material system has no influence with respect to the changes engendered by varying the mold temperature.

#### 3.3.2. Holding Pressure

**Figure 6:**
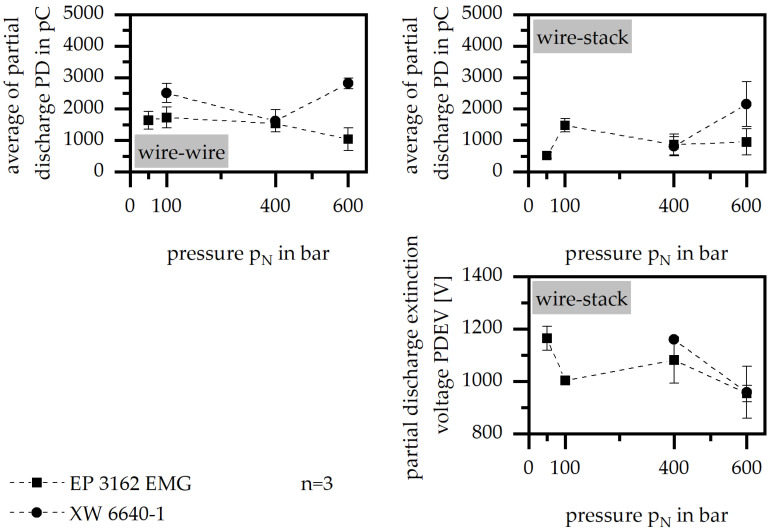
Average of partial discharge (PD) and partial discharge extinction voltage (PDEV), relative to the holding pressure in the insulation system of wire to wire and wire to stack.

The holding pressure influences the insulation system with respect to the material system, as shown in Figure 6. The PD is reduced with the increasing holding pressure in terms of the material EP 3162 EMG, but increases in terms of XW 6640-1. Further, PDEV is increased by a low holding pressure. It has to be taken into account that, in case of XW 6640-1, it was not possible to fabricate test samples with a low holding pressure that could be analyzed. It can be assumed that the holding pressure improves the insulation system by ensuring that the material is compact with a high density. This avoids partial discharges due to voids and pores. However, this means that the material itself must be able to be placed between the wire and the stack, or rather the wire and the wire. Therefore, the improvement in the insulation system owing to a high holding pressure can only be used if the general flowability of the material is guaranteed so that the material can cover the wires.

#### 3.3.3. Heating Time

**Figure 7:**
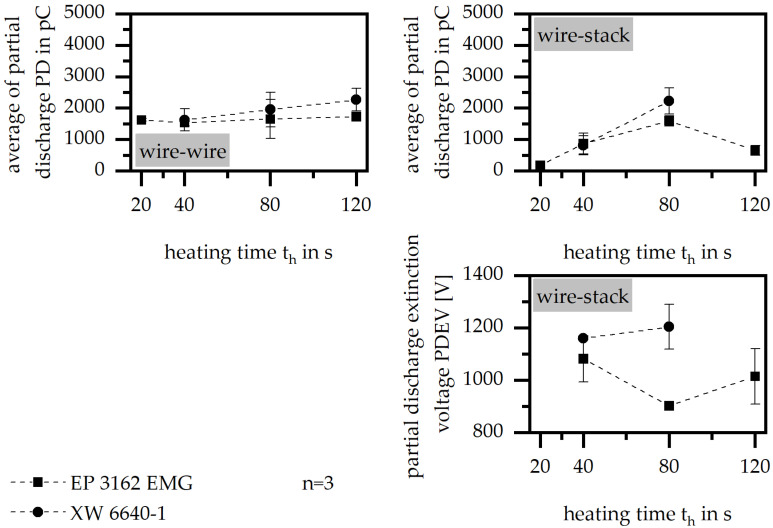
Average of partial discharge (PD) and partial discharge extinction voltage (PDEV), relative to the heating time in the insulation system of wire to wire and wire to stack.

An increase in the heating time reduces the insulation behavior in terms of the PD of the single slot sample, with only a small difference between the two material systems. While the measurement between the wires shows only minor differences, the measurement of the wire to the stack reaches an optimum for both, in terms of the high and low heating times. Therefore, the measurement between the wires represents the electrical properties of the PAI-varnish prior to the thermoset mold. Further, as shown in Figure 7, PDEV can be improved by a low and a high-value of heating time in terms of EP 3162 EMG, which might agree with the lower pot life of this material compared to XY 6640-1. Again, the process conditions have to be chosen in terms of the improvement in the flowability, to ensure an encapsulation of the wires. A low value in the heating time might result in a lack of curing, which means that this parameter has to be carefully chosen to ensure good insulation and sufficient curing. However, once the inert temperature is quite high, the sample still has a temperature above 150 °C after leaving the cavity. As cooling takes place at room temperature, further curing is likely to occur outside of the cavity, which reduces the relevance of the heating time as an influencing factor.

#### 3.3.4. Injection velocity

**Figure 8:**
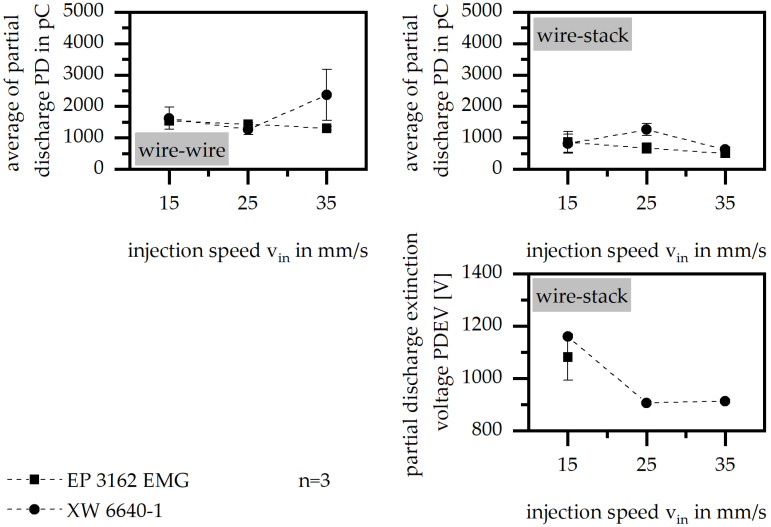
Average of partial discharge (PD) and partial discharge extinction voltage (PDEV) relative to the injection velocity in the insulation system of wire to wire and wire to stack.

The injection velocity has only a little effect on PD, where the reduction in the injection velocity generally improves PD independent of the material system. PDEV is highly effected by the injection velocity, as shown in Figure 8, where the value of PDEV is significantly reduced by higher velocities in terms of XW 6640-1; a sample fabrication is not possible in terms of EP 3162 EMG. This reduction and lack of fabrication is in accordance with the heating of the material due to shearing, which reduces the flowability of the material massively. As EP 3162 EMG reveals a high increase in the viscosity within a small range in the temperature, the higher injection velocity does not allow the fabrication of samples. In terms of the material XW 6640-1, the viscosity reaches a small value over a long period of temperature exposure so that samples can be fabricated, but the insulation is insufficient, which coincides with low values of PDEV.

#### 3.3.5. Slot Depth

**Figure 9:**
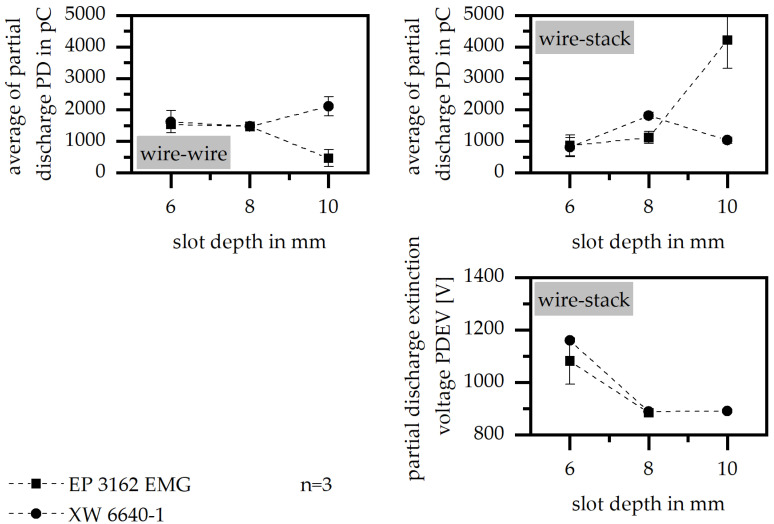
Average of partial discharge (PD) and partial discharge extinction voltage (PDEV) relative to the slot depth in the insulation system of wire to wire and wire to stack.

The variation in the slot depth improves the electric properties in terms of the PD between the two wires with increasing depth. It is assumed that the increasing space between the two wires within the slot means that there is a higher possibility of placing the insulation material not only between the wire and the stack, but also between the two wires. Further, the wider slot cross sections allow the higher displacement of the conductors relative to each other. The smaller overlap in the conductors and the additional insulation of the molding compounds prevent partial discharges more severely. The value of PD between the wire and the stack is further improved due to a higher slot depth in terms of the material XW 6640-1; however, this is reduced in terms of EP 3162 EMG, as shown in Figure 9. The increasing value of the slot depth further reduces PDEV for both materials, so that the higher slot depth has only a positive impact on PD in terms of the electric properties.

#### 3.3.6. Flow-Improving Grooves

**Figure 10:**
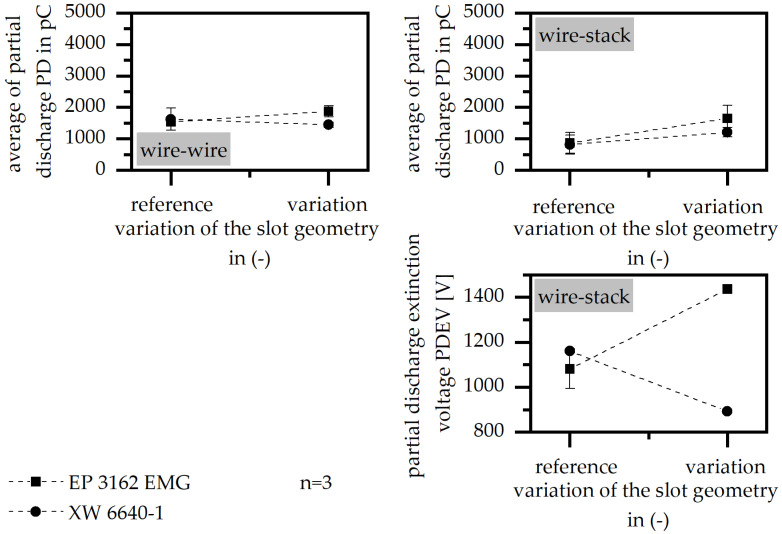
Average of partial discharge (PD) and partial discharge extinction voltage (PDEV), relative to the variation in the slot geometry and flow-improving grooves in the variation in the insulation system of wire to wire and wire to stack.

The variation in the slot geometry in terms of flow-improving grooves has only a little impact on the PD level, but improves PDEV significantly for the material EP 3162 EMG, as shown in Figure 10. It can be assumed that the implementation of the grooves changes the flow conditions, which has a positive impact in terms of the material EP 3162 EMG; this material sees a sharp increase in the viscosity with respect to the temperature and is, therefore, more sensitive in terms of flow-improving conditions. As the viscosity of XW 6640-1 reveals a smoother increase in the viscosity relative to the temperature, the impact of the flow-improving grooves is less.

With respect to the influence of the process parameters and the slot geometry on the insulation system, Table 2 summarizes the impact of the parameters and shows the possible values in terms of an improved insulation. With respect to the process parameters, a low value of viscosity and a reduced time sensitivity should be implemented to improve the possibility of encapsulating the wires, in order to increase the properties of the insulation system between the wire and the stack, as well as between the wires. The improvement in the electric properties can be further improved by an increasing slot depth or the implementation of flow-improving grooves; this is in order to increase the flow conditions and the space between the wires and the wire, as well as between the wire and the stack.

### 3.4. Microscopy

In addition to the characterization of the average of the partial discharge (PD) and the partial discharge extinction voltage (PDEV), microscopy images of the slot were analyzed to further assess the behavior of the single slot sample in terms of the insulation system. The air space between the two wires and the general position of the wires in between the stack was defined with respect to the influence of the different process parameters and the variation in the slot geometry. The air space should be as low as possible and homogenous along the flow path, whereas the wires should be placed centered within the stack and covered fully from all sides with resin. The following section depicts the influence of each parameter on the encapsulation of the wires in detail. Further, the possibilities of improving the encapsulation and with that the insulation system is summarized afterwards.

#### 3.4.1. Mold Temperature

**Figure 11:**
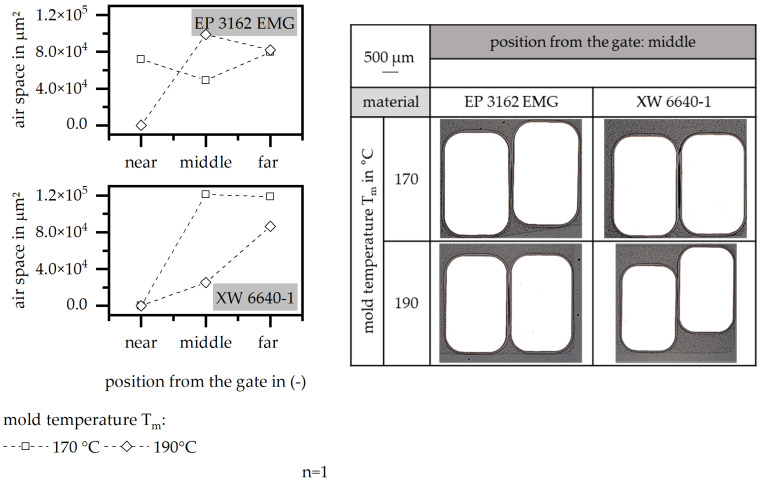
Airspace and position of the wire relative to the stack, relative to the mold temperature in the insulation system.

To define the improvement in the encapsulation by different mold temperatures, the air space and the position of the wire between the stack is shown in Figure 11. The air space is fully reduced in terms of EP 3162 EMG in the beginning of the flow path with a high mold temperature of 190 °C. However, it reaches a more homogenous behavior along the flow path with a lower mold temperature of 170 °C. For the material XW 6640-1, the air space along the flow path is more homogenous in terms of the high mold temperature, but is fully reduced for both temperature settings at the beginning of the flow path. The position of the wires in the stack shows a full encapsulation but there is a shift towards the back side of the stack, which probably causes the reduction in PD and PDEV values. Therefore, the improvement in the insulation system can be achieved by a high mold temperature as long as the position of the wires throughout the flow path can be fix in a defined way so that the shift of the wires to the back of the stack can be prevented.

#### 3.4.2. Holding Pressure

**Figure 12:**
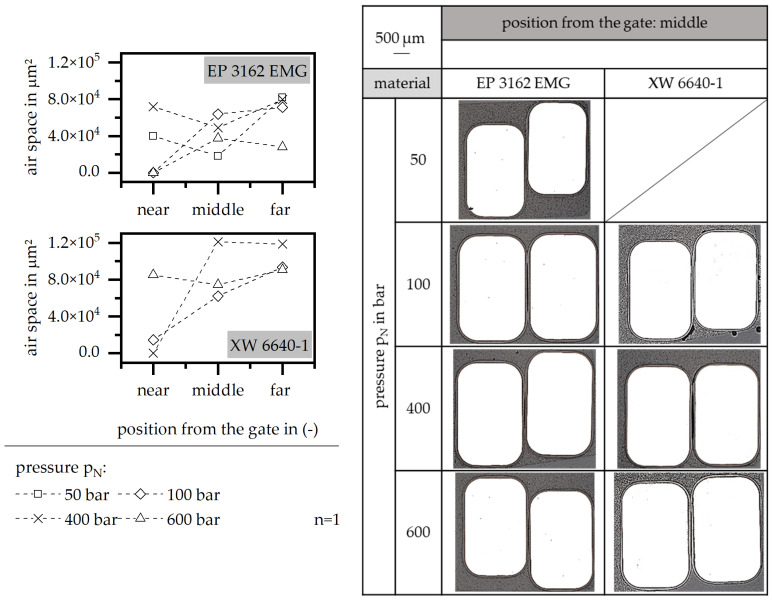
Airspace and position of the wire relative to the stack, relative to the holding pressure in the insulation system.

The influence of the holding pressure on the encapsulation of the wires is shown in Figure 12. In terms of the material EP 3162 EMG, a reduced air space can be reached by a high holding pressure of 600 bar. Lower values reduce the uniformity of the air space along the flow path. In terms of the material XW 6640-1, a high holding pressure realizes a homogenous air space along the flow path with a higher value compared to EP 3162 EMG. This is in accordance with the characterization of PD and PDEV. The high holding pressure might force the wires into one corner of the stack, which means that the encapsulation cannot be fully realized unless the position of the wires relative to the stack is defined at the beginning of the injection molding process.

#### 3.4.3. Heating Time

**Figure 13:**
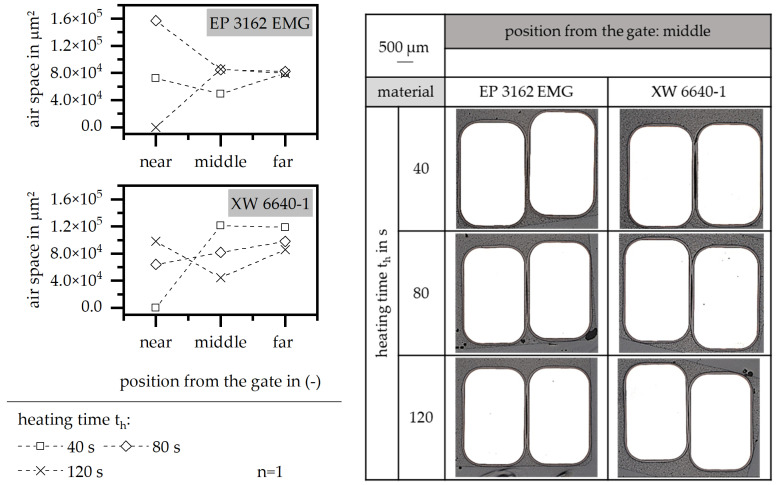
Airspace and position of the wire relative to the stack, relative to the heating time in the insulation system.

Figure 13 shows the impact of the heating time on the encapsulation of the wires in terms of the air space and the position of the wires relative to the stack. Both materials reveal a homogenous air space with a reduced heating time, whereas the value depends on the material behavior and especially on the viscosity route relative to time and temperature. This means that the air space is improved with a heating time of 40 s in terms of EP 3162 EMG and of 80 s in terms XW 6640-1. This second material has less sensitivity regarding the increase in the viscosity with the increasing temperature and time, which means that a longer heating time is necessary in order to ensure a proper curing process and the defined position of the wires. This can cause the distortion of the wires within the slot, especially in terms of XW 6640-1.

#### 3.4.4. Injection Velocity

**Figure 14:**
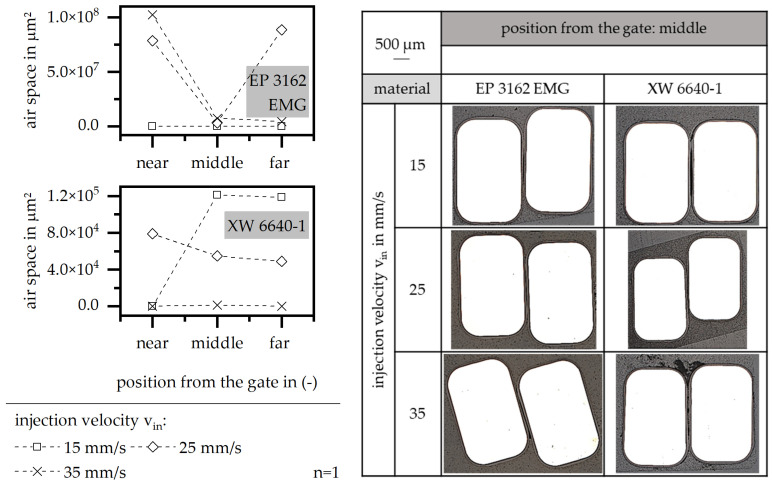
Airspace and position of the wire relative to the stack, relative to the injection velocity in the insulation system.

In order to define the improvement in the encapsulation due to different injection velocities, the air space and the position of the wire between the stack are shown in Figure 14. The air space is fully reduced along the flow path in terms of EP 3162 EMG, with a low value in the injection velocity. Further, a high value also realizes a low air space between the wires in the case of EP 3162 EMG, but the space is highly increased at the beginning of the flow path. In terms of the material XW 6640-1, the air space can be fully reduced along the flow path in terms of a high value in the injection velocity. However, the microscopy image reveals a great air bubble in this case. This means that the behavior of PD and PDEV, in case of a high injection velocity for the material XW 6640-1, can be further increased if this trapped air is removed, for example, in case of a higher holding pressure. In terms of the material EP 3162 EMG, the reduced injection velocity is more suitable with respect to the material behavior along the whole flow path, as the increase in the heat throughout creates shear forces, which is in accordance with a high injection velocity, hinders a full encapsulation along the flow path and makes twists of the wire more likely.

#### 3.4.5. Slot Depth

**Figure 15:**
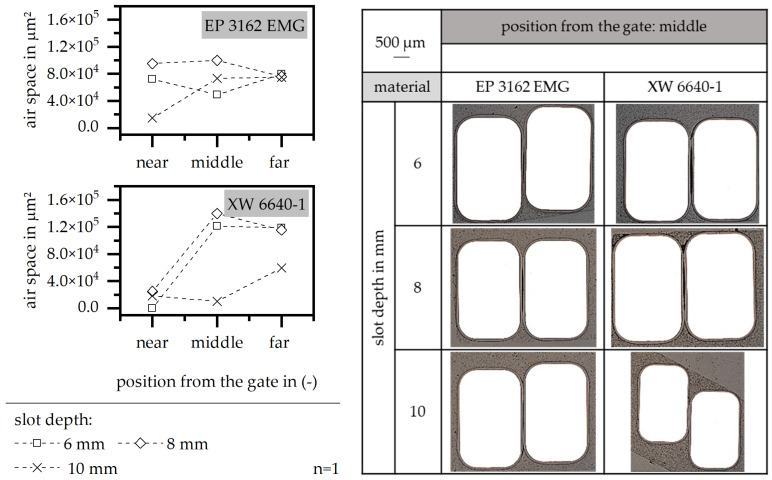
Airspace and position of the wire relative to the stack, relative to the slot depth in the insulation system.

The air space between the wires can be reduced with an increasing slot depth, as shown in Figure 15. This effect is strongly realized in terms of the material XW 6640-1, where the value of the space between the wires in reduced throughout the whole flow path. In terms of the material EP 3162 EMG, the reduction in the air space is only significant at the beginning of the flow path near the gate and reaches a constant level in the rest of the flow path. The wires are fully covered with resin but it is more likely that the wires are forced to a corner of the cavity or are twisted within the slot as the slot depth increases. This means that the increase in the slot depth improves the conditions of the full encapsulation of the wires in the slot, but makes a local fixation of the wires more necessary in order to prevent the wires from shifting to one edge of the slot or twisting.

#### 3.4.6. Flow-Improving Grooves

**Figure 16:**
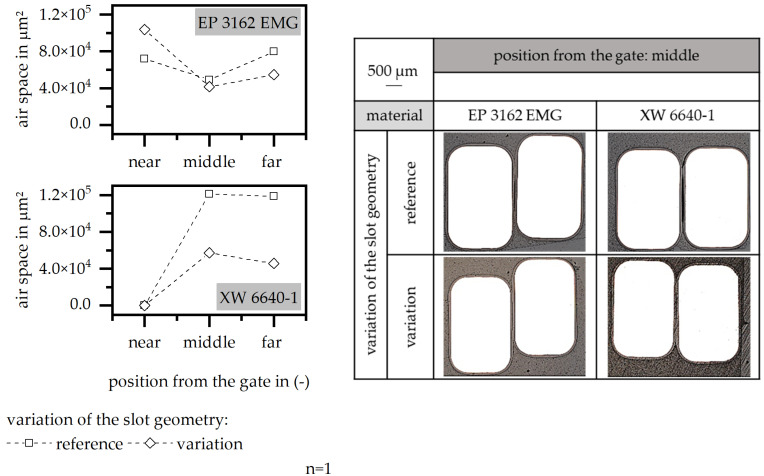
Airspace and position of the wire relative to the stack, relative to the variation in the slot geometry and flow-improving grooves in the insulation system.

The variation in the slot geometry via the implementation of flow-improving grooves reduces the air space between the wires throughout the flow path. This effect is stronger in terms of the material XW 6640-1. Both materials reach a homogenous level of air space along the flow path, as shown in Figure 16. There is no significant change in the encapsulation of the wires present, as the wires are covered with resin but tend to be placed in one edge of the cavity. This shift of the wires in the cavity is not changed throughout the flow-improving grooves. Nevertheless, the implementation of the flow-improving grooves has a positive effect on the air space between the wires by reducing this value and reaching a homogenous level along the flow path.

With respect to the influence of the process parameters and the slot geometry on the encapsulation of the wires, Table 3 summarizes the impact of the parameters and shows the possible values in terms of an improved insulation. Relative to the impact on the electric properties, which are summarized in Table 2, the improved conditions in terms of the electric properties and the full encapsulation differ in some parameters. With respect to the slot geometry, the improved conditions are similar, and both the electric properties and the full encapsulation can be reached by an increase in the slot depth or the implementation of the flow-improving grooves. Further, in terms of the process parameters, the electric properties and a full encapsulation can be enhanced by an increase in the holding pressure and a reduction in the heating time. Different suggestions, in terms of the improvement in the conditions, are reached by the mold temperature and the injection speed. The different improvements in terms of the mold temperature are based on the shift of the wires in the slot; this could be reduced by the fixation of the wires. Further, the difference with respect to the injection speed is in accordance with the time and temperature-dependent viscosity behavior of the two materials.

## 4. Conclusions

Based on the investigations of this paper, two different options that can be used in order to improve the insulation systems of electric drives by injection molding were found. These main routes which were analyzed by focusing on the process conditions on the one hand, and on the geometry of the slot on the other hand. The evaluation of the different parameters was realized in terms of the electric properties and the full encapsulation by microscopy images. In between the two conditions, the recommendations of the parameters were mainly similar. Further, the main factor was the viscosity of the resin with respect to time and temperature, which was shown to have a massive impact on the insulation of the wires and thus on the electric properties. Therefore, the conditions were chosen in terms of the improvement in the viscosity; therefore, the encapsulation was more likely to occur and thus an improvement in the electric properties could be realized. In addition to the viscosity, the process parameters and the slot geometry also had an impact on the position of the wires within the stack or slot. The conditions had to be chosen in such a way that a twist in the wires or a shift towards the edge of the slot was hindered.

Further investigations plan to transfer the conditions of the processing operation to full stator geometry, in order to realize the use of an insulation system in an application. The main influencing factors, which were investigated within this paper, should be optimized in order to ensure the suitability of using the stator in an application. Therefore, it is essential that the displacement of the wires in the slot is prevented, in order to ensure a stable insulation process. In addition, the influence of the material system and—in more detail—of the filler system, should be investigated by placing a defined amount of filler in a pure EP resin.

## Figures and Tables

**Table 1:** Processing parameters of injection molding to fabricate the single slot sample [reference: column 0|--: lowest value|-: low value|+: high value|++: highest value].

Process Parameters	Niveau of the Processing Conditions				
	--	-	0	+	++
mass temperature [°C]	x	x	85	x	x
mold temperature [°C]	x	x	170	190	x
insert temperature [°C]	x	x	170	x	x
holding pressure [bar]	50	100	400	600	x
heating time [s]	x	x	40	80	120
injection speed [mm/s]	x	x	15	25	35

**Table 2:** Improvement in PD and PDEV in the insulation system of the single slot sample by process conditions and slot geometry.

		Improvement of PD and PDEV by
Process parameters	mold temperature	reduction
	holding pressure	increase
	heating time	reduction
	injection speed	reduction
Slot geometry	slot depth	increase
	flow-improving grooves	implementation of the variation

**Table 3:** Improvement in full encapsulation of the wires within the stack in the insulation system of the single slot sample by process conditions and slot geometry.

		Improvement in Full Encapsulation by
Process parameters	mold temperature	increase
	holding pressure	increase
	heating time	reduction
	injection speed	reduction/increase ^(*)^
Slot geometry	slot depth	increase
	Flow-improving grooves	implementation of the variation

^(*)^: relative to time and temperature-dependent viscosity route of the material.

## Data Availability

Restrictions apply to the availability of these data. Data is available with the permission of the author.

## References

[1] Gläßel T., Franke J. (2017). Kontaktierung von Antrieben für die Elektromobilität. Z. Wirtsch. Fabr..

[2] Küchler A. (2017). Hochspannungstechnik: Grundlagen—Technologie—Anwendung.

[3] Riedel A., Masuch M., Weigelt T., Gläßel T., Kühl A., Reinstein S., Franke J. Challenges of the Hairpin Technology for production Techniques. Proceedings of the 2028 21st International Conference on Electrical Machnies and Systems (ICEMS).

[4] Tzscheutschler R., Olbrisch H., Jordan W. (1990). Technologie des Elektromaschinenbaus.

[5] (2008). Polymers for Electrical Insulation: Coatings and Casting Materials for the Electrical Industry.

[6] Riedel A., Kneidl M., Seefried J., Kuehl A., Franke J., Behrens B.-A., Brosius A., Drossel W.-G., Hintze W., Ihlenfeldt S., Nyhuis P. (2022). Comparison of Various Manufacturing Processes for Hairpin-Stators with Different Conductor Material. Production at the Leading Edge of Technology.

[7] Baur E., Osswald T., Rudolph N., Brinkmann S., Schmachtenberg E. (2013). Saechtling Kunststoff Taschenbuch.

[8] Brahm M. (2009). Polymerchemie Kompakt: Grundlagen—Struktur der Makromoleküle. Technisch Wichtige Polymere und Reaktionssysteme.

[9] Woebcken W. (1988). Kunststoff-Handbuch: 10: Duroplast.

[10] Mayer C. (2000). Prozessanalyse und Modellbildung zur Herstellung Gewebeverstärkter, Thermoplastischer Halbzeuge. Ph.D. Dissertation.

[11] Arcy H.D. (1856). Exposition et Application des Principes a Suivre des Formules a Emplyer dans les Questions de Distribution d’eau.

[12] Deringer T. (2019). Integrative manufacturing of thermoset injection molding components with continous fiber-reinforcement. Z. Kunstst..

[13] Reuter S., Sorg T., Liebertseder J., Dopperbauer M. Design and Evaluation of a houseless high Performance Machine with thermoset Molded internal Cooling. Proceedings of the 2021 11th IEEE International Electric Drives Production Conference (EDPC).

[14] Szabo L., Simon F., Lambert Z., Szalay P. (2015). Stator Having an Extrusion Coating and Electrical Machine with the Stator.

[15] Ortloff P. (2008). DEvice for Controlling Direct Current Motor, Has Coil Elements, Which Are Arranged Partially in Star-Connection with Different Windings and Has Neutral Point, Where Windings and Neutral Point Are Connected to a Computer Unit.

[16] Rösel U., Kneidl M., Drummer D., Franke J. (2022). Possibilities of Integrated Fabrication of Insulation Systems in Electric Drives by Injection Molding of Thermosets. Polymers.

[17] (2019). Raschig GmbH, Technisches Datenblatt: Epoxidur EP 3162 E. https://www.raschig.de/downloads/buk/tdb/TDB%20-%20Epoxidur%20EP%203162%20E.pdf.

[18] (2022). Duresco GmbH. Technisches Datenblatt: XW 6640-1. https://www.duresco.ch/produkte/datenblaetter/.

